# Diet in Disease

**Published:** 1892-11-19

**Authors:** Ernest Hart

**Affiliations:** Bachelier-ès-Sciences-es-Lettres (rèstreint), formerly Student of the Faculty of Medicine of Paris, and of the London School of Medicine for Women


### DIET IN DISEASE.

By Mrs. Ernest Hart, Bachelier-ès-Sciences-es-Lettres (rêstreint), formerly Student of the Faculty of Medicine of Paris, and of the London School of Medicine for Women.

### VI.—RESTORATIVES.

### Coffee—Cocoa—Chocolate.

Coffee is a valuable restorative, which though it closely resembles tea in constitution, has its own special characteristics and properties. From time immemorial coffee has been known and valued in Arabia, the native home of the coffee plant, and the finest coffee still comes from Mocha. The M.ors and Arabs of the Orient, who are forbidden by their religion to take alcohol, find in coffee a stimulating bev rage. The first coffee-house was opened in London in 1652, and since that date the use of coffee has constantly increased, though owing, in a great measure, to the imperfect way in which it is made in England, it is not nearly so favourite a beverage here as in France.

**The Coffee Plant and Coffee Berry.**—Coffee is the seed of the fruit of the coffee-tree, a shrub-like plant which is cultivated with the greatest success in Arabia, Turkey, the West Indies, and Java. The only preparation the berries undergo is that of roasting, during which the peculiar aroma, taste, and flavour of the coffee are brought out.

**The Constituents of Coffee.**—Coffee, like tea, contains three active principles. These are the alkaloid **caffeine**, which is identical with, and has the same properties as thein; secondly, an astringent substance resembling tannin, is preseut in much smaller quantities than in tea; and thirdly, a volatile oil developed in roasting, which gives the coffee its aromatic odour. Coffee, like tea, also contains a considerable amount of gluten, which is only slightly soluble in water.

The following approximate analysis will show the differences between tea and coffee :—

| | Tea. | Coffee. |
|---|---|---|
| Water | 5 | 12 |
| Tannic acid | 15 | 5 |
| Theine | 0·50 | 0·75 |
| Gluten | 25 | 13 |
| Fat and volatile oil | 4 | 13 |
| Woody fibre, gum, &c. | 50·50 | 56·25 |
| | 100·0 | 100·0 |

From this table it will be seen how closely tea and coffee resemble one another. Tea is, however, the more astringent drink, and coffee the more stimulating and aromatic. In Europe the ground coffee berry is generally simply infused, when its thein, tannic acid, and volatile oil are dissolved in the water. Among some of the Eastern nations the custom prevails of pounding the coffee in a mortar till a fine powder is produced. The coffee grounds are left in the cup, and are swallowed with the coffee. In this way the gluten and nutritive properties of the coffee berry are consumed, and thus a cup of coffee becomes a very nutritious food.

**The Physiological Effects of Coffee.**—Coffee has been called in France "an intellectual drink," owing to the fact that it has a decided stimulating influence on the nervous centres, lessens the need for sleep, and increases the capacity for mental work. It also seems to have, like coca erythroxylon, the power of augmenting the functional activity of the muscles, even while it diminishes tissue waste. Like tea, coffee lessens the sense of hunger, and will banish fatigue. To the soldier on the march it has proved the most valuable restorative, and for the explorer in the Arctic regions a warm cup of coffee has been declared to be a far better nightcap than rum and water.

**As to the power of coffee to sustain under fatigue, cold, and exposure** I may, perhaps, be allowed to give a personal illustration. Some years ago a party of travellers, including myself, started from Leukabad with the intention of climbing the Gemmi and crossing a pass involving a toilsome mountain walk of not less than thirty miles. The Gemmi is a solid wall of granite, which rises precipitously to the height of 5,000 feet, and so precipitous is this immense cliff that travellers are only allowed to mount it on foot by means of the narrow paths which are cut in zig-zags along its bare surface. We had not proceeded far on our journey when we were overtaken by a severe snowstorm. Unwilling to turn back, and enjoying the beauty of the storm, we steadily climbed to the summit of the Gemmi. Here, however, we met the full force of the storm, which was sweeping unchecked over the glacier-worn surface of the snow-covered plain. In the teeth of the wind and the blinding snow we pressed forward mile after mile, while the icicles hung from the beards of the gentlemen and the hats of the ladies. Presently we came in sight of the lonely Hospice which stands beside the black waters of a tarn. Glad of shelter from the storm we turned into the little inn and shook the snow and icicles from our clothes, and changed wet stockings and boots for dry ones, always carried with us as a precaution. "What shall we take?" was then the question, and various stimulants and hot drinks were suggested, but my husband, knowing that we did not wish to stay the afternoon and evening at the dreary inn, advised us not to touch alcohol, but to take only hot coffee if we wished to continue our journey. To the disappointment of the innkeeper, we thus ordered nothing more than a large pot of smoking hot coffee, with which we refreshed ourselves. After a short rest, and stimulated and warmed by the coffee, we again started gaily on our journey, and walked another sixteen miles through the snow-covered forests to the nearest green valley, which we reached late. We were all convinced that it was owing to the coffee that we made this toilsome journey in the snowstorm on foot, and that if we had taken alcohol as a restorative we should have spent the afternoon tired and chilly by the inn fire. This view is strongly expressed by Germain Sée, the French physician. In comparing alcohol and coffee, he says: "The muscular system and muscular energy are marvellously roused by coffee, and a man fatigued or overworked can find no more wholesome support, whereas alcohol produces in the muscles a dubious passing excitement, and in the end a degeneration of all the organs of human activity." Sir William Roberts says that coffee inhibits or slows stomach digestion, which action is very marked in strong black coffee, hence it is inadvisable for those who have weak digestions to take strong coffee after dinner.

**How to Make Good Coffee.**—Coffee is not sufficiently appreciated in this country. This is due in a great measure to the imperfect way it is made. Coffee should be purchased in small quantities fresh roasted. It should be ground just before it is used, and should be perfectly pure without any admixture of chicory. Coffee can be made either in a percolator, in which an infusion is made by slowly pouring boiling water through the ground coffee, or it may be boiled in an enamelled saucepan and then strained; or it may be prepared in any of the patent machines which boil the coffee for a limited time only. In my opinion the full flavour of the coffee is only extracted by boiling, but the time of boiling must be very short, not exceeding more than a minute. Dr. Burney Yeo suggests that the coffee grounds left in the bag of the percolator should be boiled in water, and that this water should be used to percolate the next day's coffee with. In the French army coffee is made in a machine in which the water is passed in small jets several times through the ground coffee. Good coffee cannot, however, be made without a sufficient quantity of ground coffee; not less than 2 oz. to a pint of water should be used.

The small popularity coffee has in England is due to the fact that it is largely adulterated with chicory, and also that it is made so weak that its stimulative, refreshing, and aromatic qualities are not enjoyed. It can be drunk much more continuously than tea without inducing bad consequences, though in some people strong coffee produces palpitation of the heart.

**Chicory** is the roasted and ground root of the wild endive. It contains a volatile oil and a bitter principle, but no caffeine. It is added to coffee to increase the flavour, and to give the appearance of strength. It readily stains water, so that its presence can at once be detected by putting a pinch of what has been purchased as ground coffee in a cup of cold water; if pure the water remains clear, if chicory is present the water is stained.

**Cocoa.**—Cocoa is derived from the seed of a plant called the theobroma cacao. The name was given by the botanist Linneus, who expressed his high opinion of cocoa by the name "theobroma," which means "food for the gods." The seeds are embedded in a pulpy fruit, when ripe they are separated, sun-dried, and roasted. Cocoa nibs consist of the crushed kernels of these seeds; but cocoa such as we generally use has undergone a long and elaborate process of preparation. The following analysis (Payen) of cocoa will show in what way it differs from tea and coffee:—

| | |
|---|---|
| Cacao Butter ... ... ... ... | 50 |
| Albumen, Fibrine, &c. ... ... ... | 20 |
| Theobroma ... ... ... ... ... | 2 |
| Starch ... ... ... ... ... | 10 |
| Cellulose ... ... ... ... ... | 2 |
| Mineral Matter ... ... ... ... | 4 |
| Water ... ... ... ... ... | 12 |
| | 100 |

It is thus seen that cocoa is rich in fat, that tannin is absent, and that it contains an alkaloid, theobromine. This alkaloid is almost identical with theine and caffeine, and has probably the same stimulating and inhibitory action, but as it is present in diluted cocoa in very small amount, cocoa is a less stimulating drink than tea and coffee. Its constitution shows, however, that it is highly nourishing. In the various prepared cocoas, the excess of fat has been extracted, and sugar, and sometimes starch, added. There are three ways of making cocoa according to the mode in which the cocoa has been prepared. Stewing: The cocoa nibs are slowly boiled or stewed for several hours, and the subnatant fluid is poured off. A more stimulating, but less nourishing drink is obtained than from prepared cocoas. Infusing: In cocoas prepared without the admixture of starch, such as Van Houten's and Schieweitze's, it is necessary only to mix a certain amount with hot water to make a soluble infusion. These cocoas are particularly suitable to those persons who must avoid starch in their diet. Boiling: Most prepared cocoas contain a large amount of added starch, it is therefore necessary to boil them before drinking. They form a highly nutritious and fattening food, especially when taken, as is usual, with a large amount of milk.

**Chocolate** is prepared from cacao seeds by grinding, and by the addition of a large amount of sugar and various flavouring matters. It forms a luxurious and highly nutritious food, and is very rich in all the necessary constituents of diet, viz., hydro-carbons, carbo-hydrates, and albuminates. A small amount of solid chocolate will be found a most valuable preventive against hunger when circumstances oblige one to go long without food. I have frequently found that a penny slot of chocolate at a railway station will enable me, after being eight or nine hours without food, to continue my avocations without being tormented with the sense of hunger.

